# Combining circulating inflammatory proteins and thymidine kinase activity to predict survival in melanoma patients receiving immune checkpoint inhibitors

**DOI:** 10.1016/j.iotech.2026.101595

**Published:** 2026-03-19

**Authors:** S. Egyhazi Brage, F. Costa Svedman, M. Bergqvist, H. Helgadottir

**Affiliations:** 1 Department of Oncology-Pathology, Karolinska Institutet, Solna, Sweden; 2 Theme Cancer, Karolinska University Hospital, Stockholm, Sweden; 3 Biovica, Uppsala, Sweden

**Keywords:** melanoma, immunotherapy, biomarkers, inflammation proteins, thymidine kinase activity

## Abstract

**Background:**

Inflammatory plasma proteins have been associated with poorer clinical outcomes in melanoma patients treated with immune checkpoint inhibitors (ICIs). Furthermore, the plasma levels of thymidine kinase activity (TKa), an indicator of proliferation, have also been associated with adverse outcomes in several cancers. The aim of this study was to explore the relationship between TKa, systemic inflammation and survival in ICI-treated melanoma patients.

**Materials and methods:**

Fifty-eight patients with unresectable metastatic melanoma who received anti-programmed cell death protein 1 monotherapy were included in the study. Pretreatment plasma samples were analyzed for TKa levels (DiviTum® TKa assay; Biovica) and inflammatory proteins using the Olink® Target 96 Inflammation panel, and these biomarkers were correlated with patient survival.

**Results:**

A panel of five unfavorable biomarkers was selected, comprising TKa, C-X-C motif chemokine ligand 8, C-C motif chemokine ligand 3, hepatocyte growth factor and S100 calcium-binding protein A12. An increasing number of elevated biomarkers in the panel was associated with a progressively worse prognosis. In the univariate analysis, when patients with four to five unfavorable biomarkers were compared with those with none, the hazard ratio (HR) was 2.43 [95% confidence interval (CI) 0.91-6.44, *P* = 0.075] for progression-free survival (PFS) and 10.62 (95% CI 3.11-36.26, *P* < 0.001) for overall survival (OS). When adjusting for sex, age, stage and lactate dehydrogenase (LDH), the HR was 1.97 (95% CI 0.61-6.41, *P* = 0.26) for PFS and 11.03 (95% CI 2.53-48.12, *P* = 0.001) for OS.

**Conclusion:**

This multi-biomarker panel was independently associated with poor OS in ICI-treated melanoma patients. This finding suggests a prognostic value of the panel, indicating its potential relevance for stratifying patients by risk, although further validation in larger cohorts is warranted.

## Highlights

• Circulating inflammatory proteins and TKa were analyzed in patients with metastatic melanoma receiving immunotherapy.
• A subset of inflammatory proteins correlated with TKa.
• A multi-biomarker panel comprising TKa and inflammatory proteins was identified as associated with poor survival.
• An increasing number of elevated unfavorable biomarkers was associated with a gradual decline in PFS and OS.
• The multi-biomarker panel sustained as an independent predictor of OS after adjusting for sex, age, LDH and stage.

## Introduction

The introduction of immune checkpoint inhibitors (ICIs) for the treatment of advanced melanoma has significantly prolonged survival outcomes for many patients.^1^^,^^2^ Despite this success, there are still many patients who do not benefit from this treatment, and an accurate and reliable method of identifying likely responders remains elusive.

ICIs, particularly those targeting programmed cell death protein 1 (PD-1) and its ligand programmed death-ligand 1 (PD-L1), create antitumor immune responses by disrupting inhibitory signaling in T cells. Treatment response is influenced by multiple biological factors, including tumor genetics, immune cell infiltration and systemic immune signaling.^3^ Among these, inflammatory responses and proliferative activity have emerged as key determinants of therapeutic outcomes. The development of better tools to predict therapy efficacy and clinical outcome in patients with advanced melanoma remains an ongoing challenge.

Previous studies have shown that elevated baseline levels of certain circulating inflammatory proteins are associated with poorer clinical outcomes in patients with metastatic melanoma treated with ICIs.^4^, ^5^, ^6^, ^7^ Similarly, the cytosolic enzyme thymidine kinase 1 activity (TKa)—a surrogate marker of cell proliferation and fundamental in the DNA synthesis—has been linked to disease stage, prognosis and treatment response in various cancers.^8^^,^^9^ As proliferating cells release TK into the bloodstream, its activity (TKa) can be measured in plasma. Furthermore, the plasma levels and dynamics of TKa have also been associated with anti-PD-1 treatment efficacy and patient outcome in advanced melanoma.^10^^,^^11^

Given the mechanistic relevance of both inflammation and proliferation to cancer progression and immune modulation, these biomarkers represent a compelling combination for patient risk stratification.

The aim of the present study was to explore whether integrating plasma TKa levels with inflammatory proteins could enhance prediction of therapy efficacy and survival outcomes in melanoma patients receiving anti-PD-1 therapy. By combining molecular indicators of proliferation and systemic inflammation, we sought to identify a biomarker signature capable of distinguishing patients with favorable versus unfavorable prognosis before treatment initiation.

## Materials and methods

### Patients

This study included 58 patients with metastatic melanoma treated with single-agent anti-PD-1 therapy at Karolinska University Hospital in Stockholm, Sweden between 2015 and 2019. Pretreatment plasma samples were collected before starting with anti-PD-1 therapy and stored at −80°C until analysis. Clinical information was collected, including age, sex, stage according to the American Joint Committee on Cancer staging eighth edition, Eastern Cooperative Oncology Group (ECOG) performance status, baseline lactate dehydrogenase (LDH) level, type and line of ICI (Table 1).^7^ Clinical visits and radiological evaluations were typically carried out every 3 months.

**Table 1:** Summary of baseline clinical characteristics and therapy regime and line of treatment

Patients	*N* = 58
Gender, *n* (%)	
Female	20 (34.5)
Male	38 (65.5)
Age, median (range)	70 (31-84)
Performance status, *n* (%)	
ECOG 0	52 (89.7)
ECOG 1	6 (10.3)
M stage, *n* (%)	
M1a	21 (36.2)
M1b	15 (25.9)
M1c	16 (27.6)
M1d	6 (10.3)
LDH, *n* (%)	
Normal	27 (46.6)
Elevated	30 (51.7)
Not available	1 (1.7)
Treatment^a^^,^^b^, *n* (%)	
Nivolumab	36 (62.1)
Pembrolizumab	22 (37.9)
Previous lines of treatment, *n* (%)	
0	57 (98.3)
≥1	1 (1.7)

ECOG, Eastern Cooperative Oncology Group; LDH, lactate dehydrogenase.

a Six patients were treated within the clinical trial NCT02752074. Three of them received pembrolizumab plus epacadostat.

b Pembrolizumab + epacadostat was considered as a single therapy since epacadostat had no effect.

All procedures were carried out in compliance with relevant laws and institutional guidelines, and the study has been approved by the Swedish Ethical Review Authority (Dnr: 2011/1980-31/1; 18 January 2012; 2021-05190; 5 December 2021). Written informed consent was obtained from all study participants.

### TKa assay

Pretreatment plasma TKa levels in plasma samples were analyzed in accordance with the manufacturer’s instructions using the Food and Drug Administration-cleared and Conformité Européenne-marked DiviTum® TKa assay (Biovica, Sweden), as previously described.^10^^,^^11^ An established TKa cut-off of 60 Du/l was used to stratify patients into low and high TKa groups.

### Olink® Target 96 Inflammation panel

Low-abundance inflammatory protein levels in plasma were recently assessed using the Proximity Extension Assay technology. Of the 92 inflammatory proteins included in the Olink® Target 96 Inflammation panel, 80 remained for analysis after discarding proteins, with >50% of the samples below Olink’s predefined limit of detection.^7^ These proteins were compared with TKa levels to identify differentially expressed inflammatory proteins between the high and the low TKa groups using the Qlucore Omics Explorer (Qlucore, Lund, Sweden) version 3.9 software. Multiple testing correction was carried out using the Benjamini–Hochberg method. Receiver operating characteristic curve analyses were carried out to determine an optimal cut-off for each inflammatory biomarker to stratify the patients into low and high protein expression groups of C-X-C motif chemokine ligand 8 (CXCL8), C-C motif chemokine ligand 3 (CCL3), hepatocyte growth factor (HGF) and S100 calcium-binding protein A12 (S100A12). The most optimal sensitivity and specificity in predicting 24 months of OS, using the Youden index (sensitivity + specificity − 1) was chosen to determine suitable cut-offs. In addition, we applied an alternative analytical approach to choose the cut-offs based on median split.

### Statistical analysis

Survival analyses were carried out to assess the prognostic value of combined biomarkers. The Kaplan–Meier method was used to estimate progression-free survival (PFS) and overall survival (OS), and survival curves were compared using the log-rank test of trend. Univariate and multivariate Cox regression analyses were used to evaluate the effect of different variables on clinical outcomes. The PFS time was calculated from the date of treatment start until the date of disease progression or death, whichever came first, or last follow-up. The OS time was calculated from the date of treatment start until the date of death or last follow-up. All analyses were done using the statistical software GraphPad Prism (GraphPad Software, Boston, USA) version 9.5.1 and STATA (StataCorp LLC, College Station, USA) version 19. Statistical significance was defined as *P* value < 0.05.

## Results

### Baseline clinical characteristics

Fifty-eight patients with metastatic melanoma were included in the study. The median age was 70 years (range 31-84 years) and 65.5% were male. A majority of the patients 52/58 (89.7%) had an ECOG performance status of 0. Twenty-one patients had tumor stages M1a (36.2%), 15 M1b (25.9%), 16 M1c (27.6%) and 6 M1d (10.3%). Elevated LDH was detected in 51.7% of the patients, but LDH elevations were modest overall, with only 2 of 58 patients exceeding > 2 × upper limit of normal. All except one patient received anti-PD-1 monotherapy as first-line therapy. Clinical characteristics are summarized in Table 1.

### Correlation of inflammatory proteins with TKa levels

**Figure 1:**
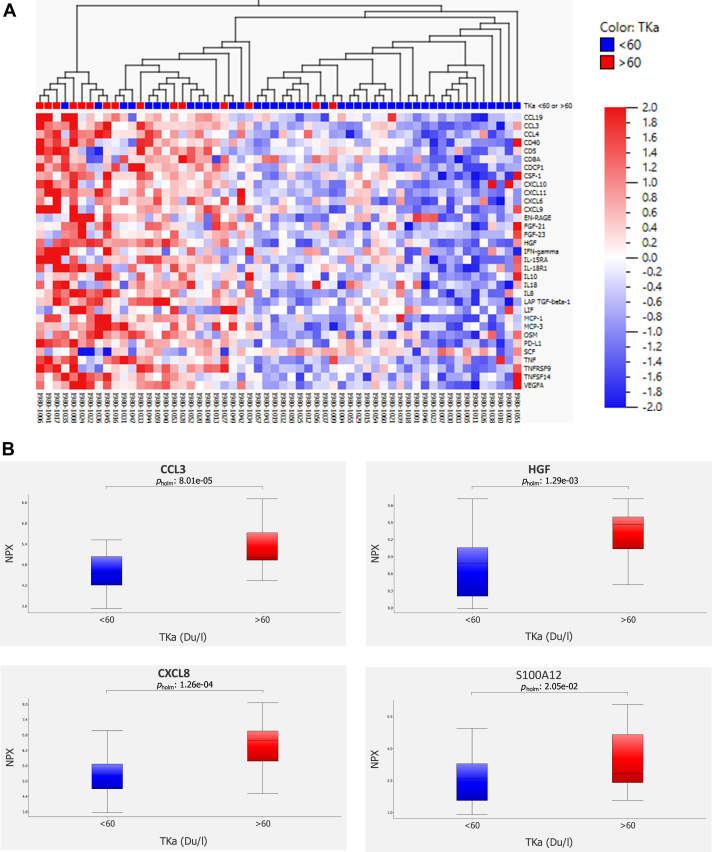
**Correlation of inflammatory proteins with TKa levels.** (A) Heatmap showing 33 differentially expressed inflammatory proteins between patients with low (blue) and high (red) TKa levels (log2 values). (B) Box plots showing expression of CCL3 (adjusted *P* = 8.01e^−05^), HGF (adjusted *P* = 1.29e^−03^), CXCL8 (adjusted *P* = 1.26e^−04^) and S100A12 (adjusted *P* = 2.05e^−02^) in patients with low or high TKa levels. Multiple testing correction was carried out using the Benjamini–Hochberg (A) and Holm–Bonferroni (B) methods, respectively. CCL3, C-C motif chemokine ligand 3; CXCL8, C-X-C motif chemokine ligand 8; HGF, hepatocyte growth factor; NPX, normalized protein expression; S100A12, S100 calcium-binding protein A12; TKa, thymidine kinase activity.

From the Olink® Target 96 Inflammation panel, 80 circulating inflammatory proteins were included in the analysis, whereof 33 showed significant differential expression (adjusted *P* value < 0.05) between patients with low and high TKa levels (Figure 1A, Supplementary Table S1, available at https://doi.org/10.1016/j.iotech.2026.101595). Notably, CXCL8, CCL3, HGF and S100A12—each previously associated with poor prognosis^7^—were enriched in the high TKa group (>60 Du/l) (Figure 1B). None of the other 29 circulating inflammatory proteins were associated with worse prognosis in this cohort when adjusted for multiple testing.^7^ Based on these findings, a five-biomarker panel was established, comprising TKa and the four inflammatory proteins (CXCL8, CCL3, HGF and S100A12). High expression of any of the four inflammatory proteins or high TKa were considered unfavorable. We investigated whether the combination of these five unfavorable biomarkers could improve the accuracy of outcome prediction.

### Association of increasing number of elevated unfavorable biomarkers with shorter PFS and OS

The first analysis was conducted to show the individual effect of each biomarker on survival outcome (Supplementary Figures S1 and S2, available at https://doi.org/10.1016/j.iotech.2026.101595). Although a trend toward a correlation between PFS and levels of individual inflammatory biomarkers was observed, it did not reach statistical significance, while all individual biomarkers showed a significant association with OS, except for TKa that did not reach statistical significance (*P* = 0.072).

**Figure 2:**
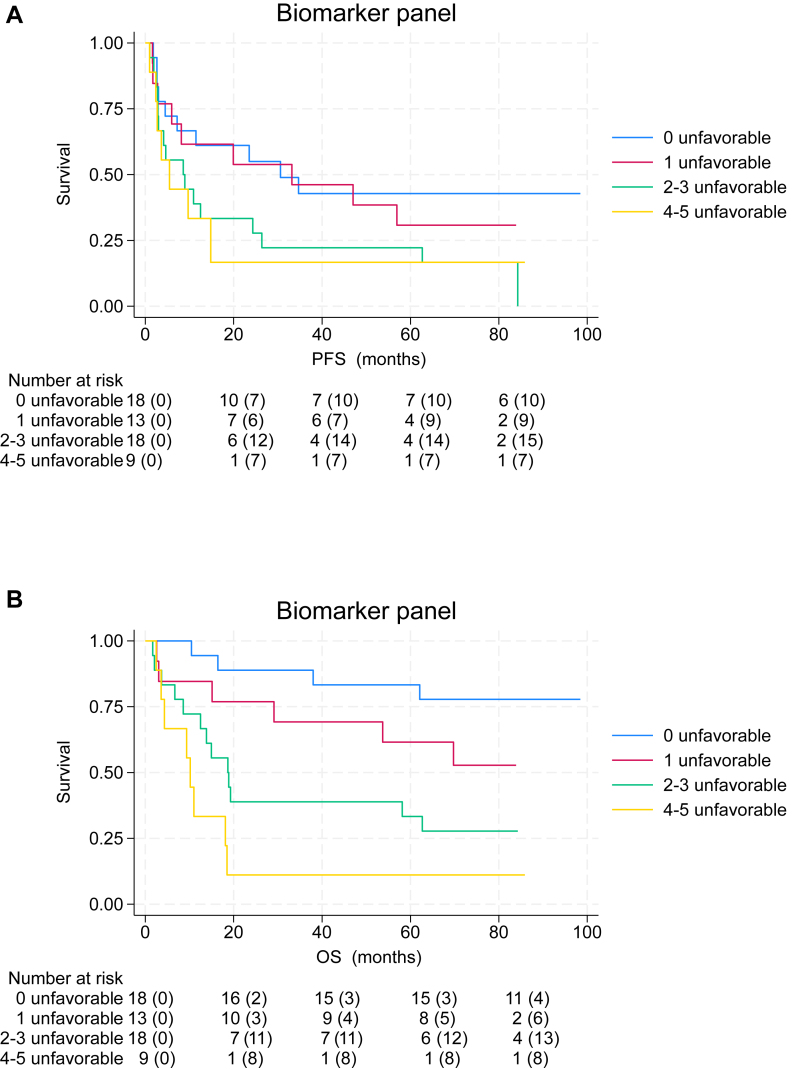
**An increasing number of elevated unfavorable biomarkers was associated with worse clinical outcome.** Kaplan–Meier curves demonstrating a pronounced gradual decrease in the proportion of (A) progression-free survivors and (B) alive melanoma patients with an increasing number of elevated unfavorable biomarkers. Number in parentheses represents the number of events for each subgroup. OS, overall survival; PFS, progression-free survival.

We then proceeded to evaluate the five-biomarker panel to determine whether it improved risk stratification beyond individual biomarkers. Median PFS was longer in patients with zero or one elevated unfavorable biomarker (30.6 and 33.2 months) than in patients with two to three and four to five elevated unfavorable biomarkers (8.6 and 5.4 months) (Figure 2A; log-rank test for trend; *P* = 0.024). The 5-year PFS rates were 43%, 31%, 22% and 11% in patients with zero, one, two to three and four to five elevated unfavorable biomarkers, respectively (Supplementary Table S2, available at https://doi.org/10.1016/j.iotech.2026.101595). Given the potential risk of overestimating the discriminatory performance when relying solely on the Youden index, we additionally applied an alternative analytical approach to demonstrate the robustness of our findings. The alternative analytical approach yielded PFS estimates similar to those derived from the primary method, underscoring the robustness and reproducibility of the results (the 5-year PFS rates were 38%, 46%, 22% and 16%, respectively).

In line with the PFS data, patients with zero or one elevated unfavorable biomarker exhibited higher response rates (72% and 77%, respectively) than those with two to three or four to five elevated biomarkers (both 56%) (Supplementary Figure S3, available at https://doi.org/10.1016/j.iotech.2026.101595). The association between an increasing number of elevated unfavorable biomarkers and diminished clinical benefit was most evident regarding complete responses (CR): patients with zero or one elevated unfavorable biomarker demonstrated markedly higher CR rates (50% and 30.8%, respectively), whereas those harboring two to three or four to five elevated unfavorable biomarkers showed substantially lower CR rates (5.6% and 11.1%) (Supplementary Figure S3, available at https://doi.org/10.1016/j.iotech.2026.101595).

Median OS was not reached in patients with zero or one elevated unfavorable biomarker while median OS was 18.7 and 10.2 months in patients with two to three and four to five elevated unfavorable biomarkers, respectively (Figure 2B; log-rank test for trend; *P* < 0.001). The 5-year OS rates were 83%, 62%, 33% and 11% in patients with zero, one, two to three and four to five elevated unfavorable biomarkers, respectively. The alternative analytical approach also yielded OS estimates similar to those derived from the primary method (the 5-year OS rates were 83%, 62%, 50% and 20%, respectively).

The individual biomarkers demonstrated variability in 5-year PFS rate, with high-expression groups ranging from 15.56% to 22.92% and low-expression groups from 33.16% to 35.00% (Supplementary Table S2, available at https://doi.org/10.1016/j.iotech.2026.101595). A similar pattern was observed for 5-year OS rate, where high-expression groups ranged from 22.73% to 33.33%, compared with 58.14% to 69.44% among low-expression groups (Supplementary Table S2, available at https://doi.org/10.1016/j.iotech.2026.101595). Importantly, integration of multiple biomarkers produced a more pronounced separation especially for the 5-year OS outcome rates than single-biomarker analyses, demonstrating superior discriminatory capacity when biomarkers were combined. Collectively, these findings indicate that incorporating several biomarkers simultaneously provides a more robust means of identifying patients at increased risk of poor outcomes than relying on individual biomarkers alone.

Tables 2 and 3 further demonstrate the results of univariate and multivariate analyses of PFS and OS. Altogether, there was a gradual increase in hazard ratio (HR) when adding an increasing number of biomarkers, with a steeper rise for OS. For example, in the univariate analysis, when patients with four to five elevated unfavorable biomarkers were compared with those with none, the HR was 2.43 [95% confidence interval (CI) 0.91-6.44, *P* = 0.075] for PFS and 10.62 (95% CI 3.11-36.26, *P* < 0.001) for OS. When adjusting for sex, age, stage and LDH, the HR was 1.97 (95% CI 0.61-6.41, *P* = 0.26) for PFS and 11.03 (95% CI 2.53-48.12, *P* = 0.001) for OS; the multi-biomarker panel remained as an independent prognostic factor for OS.

**Table 2:** Univariate and multivariate analyses of progression-free survival in patients with metastatic melanoma receiving anti-PD-1 using the Cox regression method

	Univariate analyses			Multivariate analyses		
	HR	95% CI	*P* value	HR	95% CI	*P* value
Sex						
Female	Reference			Reference		
Male	1.58	0.81-3.11	0.18	1.94	0.81-4.62	0.14
Age, years						
<65	Reference			Reference		
>65	1.66	0.86-3.19	0.13	1.74	0.84-3.59	0.14
M1 stage						
M1a + b	Reference			Reference		
M1c + d	1.43	0.77-2.65	0.26	1.35	0.70-2.62	0.37
LDH						
Normal	Reference			Reference		
Elevated	1.78	0.96-3.32	0.069	1.89	0.93-3.86	0.079
Biomarker panel						
0 unfavorable	Reference			Reference		
1 unfavorable	1.32	0.53-3.26	0.55	1.15	0.45-2.95	0.77
2-3 unfavorable	2.20	0.99-4.86	0.053	1.44	0.63-3.33	0.39
4-5 unfavorable	2.43	0.91-6.44	0.075	1.97	0.61-6.41	0.26

CI, confidence interval; HR, hazard ratio; LDH, lactate dehydrogenase; PD-1, programmed cell death protein 1.

**Table 3:** Univariate and multivariate analyses of overall survival in patients with metastatic melanoma receiving anti-PD-1 using the Cox regression method

	Univariate analyses			Multivariate analyses		
	HR	95% CI	*P* value	HR	95% CI	*P* value
Sex						
Female	Reference			Reference		
Male	1.18	0.55-2.50	0.67	1.81	0.64-5.08	0.26
Age, years						
<65	Reference			Reference		
>65	2.12	0.97-4.61	0.059	2.10	0.87-5.08	0.098
M1 stage						
M1a + b	Reference			Reference		
M1c + d	1.41	0.69-2.89	0.34	1.09	0.50-2.35	0.83
LDH						
Normal	Reference			Reference		
Elevated	1.64	0.80-3.35	0.18	1.38	0.58-3.32	0.47
Biomarker panel						
0 unfavorable	Reference			Reference		
1 unfavorable	2.44	0.69-8.64	0.17	2.23	0.62-8.08	0.22
2-3 unfavorable	5.19	1.68-15.98	0.004	3.76	1.15-12.28	0.028
4-5 unfavorable	10.62	3.11-36.26	<0.001	11.03	2.53-48.12	0.001

CI, confidence interval; HR, hazard ratio; LDH, lactate dehydrogenase; PD-1, programmed cell death protein 1.

## Discussion

To the best of our knowledge, this is the first study providing evidence that a multi-biomarker panel comprising the proliferation biomarker TKa and a subset of pro-inflammatory proteins (CCL3, CXCL8, S100A12 and HGF) can independently stratify patients with metastatic melanoma by risk before treatment with anti-PD-1 therapy. In the present study, TKa was used as a biological anchor to identify inflammatory proteins associated with tumor proliferative activity. This approach was chosen to capture the interplay between tumor proliferation and systemic inflammation, two processes that are both known to influence disease progression and clinical outcome. An increasing number of elevated biomarkers in the identified panel was associated with a progressively worse survival. These findings suggest that in patients with four to five elevated unfavorable biomarkers, where the median PFS was only 5.4 months and median OS was only 10.2 months, PD-1 monotherapy may be insufficient. However, this observation should be considered hypothesis-generating, as our study included only patients treated with anti-PD-1 monotherapy. It is possible that such patients could benefit from alternative strategies, such as ICI combination therapy or upfront BRAF + MEK inhibitors in patients with BRAF-mutated tumors. On the other hand, the patient group with no elevated unfavorable biomarker had a 5-year PFS rate of 39% and OS rate of 83% with anti-PD-1 monotherapy suggesting that monotherapy may be sufficient for many of these patients and thus reduce the risk for development of severe immune-related adverse events compared with ICI combination treatment.

There is a discrepancy between the strong OS association and the weaker PFS association to this multi-biomarker panel indicating that it is only of prognostic value independent of choice of treatment. However, PFS may in this real-world cohort be influenced by several factors that can impact the biological associations such as the possibility of pseudo-progression, and in some cases the continuation of treatment beyond radiologic progression can introduce noise into PFS measurements. These sources of misclassification are well-recognized limitations of real-world PFS data and may disproportionately affect analyses in smaller cohorts. In contrast, OS is less susceptible to these biases, which likely contributes to a more consistent association observed. However, still more CRs were found among the patients with no elevated biomarkers compared with those with four to five elevated unfavorable biomarkers suggesting that this multi-biomarker panel to some extent has an impact on anti-PD-1 effect.

How these findings may translate into current clinical practice, where combination ICI regimens such as nivolumab/ipilimumab and nivolumab/relatlimab are increasingly used, needs to be explored. It is important to acknowledge that our analysis was conducted only in patients treated with anti-PD-1 monotherapy, and therefore the performance and relevance of this protein panel in the context of combination ICI therapy remain unknown. Given the distinct mechanisms, efficacy profiles and toxicity patterns of combination regimens, biomarker behavior may differ, and our results should not be assumed to generalize to these settings without further validation. We highlight this as an important future direction for the research.

Both circulating CXCL8 and HGF are well-known unfavorable biomarkers and have previously been reported to be associated with lack of response to anti-PD-1 therapy,^6^^,^^12^, ^13^, ^14^, ^15^, ^16^ while S100A12, CCL3 and TKa are less studied regarding impact on ICI therapy. Although several other inflammatory proteins, including PD-L1, interferon-gamma and tumor necrosis factor, were associated with TKa levels, they were not significantly associated with survival in this cohort. This may reflect the limited sample size as well as the context-dependent roles of these cytokines in immune regulation.

TKa is cell-cycle regulated, and its expression is low in quiescent cells and high in proliferating cells. Circulating TKa levels reflect tumor aggressiveness and correlates with anti-PD-1 treatment efficacy and patient outcome.^8^, ^9^, ^10^, ^11^ Combining TKa with selected inflammation proteins can match the complex interplay between cell proliferation and pro-tumorigenic inflammation for improved precision. In the SECOMBIT study, baseline serum TKa levels efficiently predicted the outcome of patients with BRAF V600-mutated metastatic melanoma treated with different sequences of ICI and BRAF + MEK inhibitors.^17^ Results demonstrated that patients with elevated TKa belong to a poor prognosis group and appear to benefit from a sandwich approach—an 8-week induction with BRAF-MEK inhibitors before ICI. TKa is currently involved in an extensive study program addressing different potential clinical utilities as a biomarker to predict ICI efficacy and TKa early dynamic patterns, correlating with activated immune response and increased T-cell activity, for monitoring disease status and early progression.

CXCL8 belongs to the family of CXC chemokines and plays an important role in mediating inflammatory response. In addition, CXCL8 supports tumor-promoting features such as migration, invasion and angiogenesis.^18^ It can trigger signaling activity by binding to its receptors CXCR1 and CXCR2. Both baseline and early on-treatment changes of circulating CXCL8 levels have been demonstrated to be associated with lack of response to anti-PD-1 therapy in multiple cancer types.^12^ In addition, Sanmamed et al. found that serum CXCL8 protein levels correlated with CXCL8 messenger RNA expression levels in the corresponding tumor, indicating that CXCL8 tumor levels are reflected in the circulation^12^ Treatment of CD8+ T cells with CXCL8 has been shown to increase PD-1 expression in CD8+ T cells, which could be abrogated by adding a CXCR1/2 inhibitor.^19^ Inhibitors of CXCL8 and its receptors have been developed to investigate its potential as therapeutic targets. A phase I clinical trial has been initiated to study the safety of stereotactic body radiotherapy with anti-PD-1 and anti-CXCL8 (BMS-986253) for treatment of advanced solid tumors progressing on standard therapies, including melanoma (NCT04572451). The secondary objective is efficacy with an endpoint of objective response rate which will be correlated with serum CXCL8 levels.

HGF is a multi-functional cytokine in the HGF/MET signaling pathway promoting tumor cell growth and migration as well as promoting immune evasion in the tumor microenvironment. High circulating baseline HGF levels have been associated with poorer outcomes in multiple tumor types treated with ICI therapy.^6^^,^^7^^,^^14^ Rossi et al.^6^ also observed a cumulative negative effect on ICI response by combining baseline HGF with the inflammatory proteins interleukin-6 and monocyte chemotactic protein-2/C-C motif chemokine ligand 8, in line with our results. Furthermore, HGF inhibited activated CD8+ T cells, even in the presence of anti-PD-1, and lymphocyte activation was restored by adding HGF/MET inhibitors.^14^ A phase Ib/II clinical study combining ICI with MET/HGF inhibitor crizotinib in non-small-cell lung cancer showed no evidence of increased antitumor activity of the combination treatment but 42% had dose-limiting toxicity.^20^ Lin et al. demonstrated that sequential treatment with ICI and crizotinib (*n* = 11) was associated with increased severe hepatoxicity compared with crizotinib alone (*n* = 442).^21^ By combining ICI with a more selective MET inhibitor than crizotinib, which is a broad tyrosine kinase receptor inhibitor, it may offer better tolerability.

CCL3 plays a role in inflammatory responses through binding to the receptors CCR1, CCR4 and CCR5. CCL3 is a cytokine that has been suggested as an adverse prognostic factor in hematological and solid malignancies.^22^^,^^23^ Moreover, a correlation between CCL3 and angiogenic cytokines has been found.^24^ The CCR1-CCL3 signaling pathway was proposed to be an important mediator of ICI resistance in melanoma.^25^ The combination of a CCR1 antagonist and PD-1 blockade was demonstrated to suppress tumor progression in a melanoma model.

S100A12 belongs to the S100 family of calcium-binding proteins and is proposed to be involved in systemic inflammation and autoimmune diseases.^26^^,^^27^ Many of the S100 family members are implicated in tumorigenesis and progression.^28^^,^^29^ S100A12 is abundantly expressed in and secreted by neutrophils and may thus be a surrogate marker of active neutrophils.^30^ Higher neutrophil-to-lymphocyte ratio is significantly associated with poorer clinical outcome among patients receiving ICI therapy across multiple cancer types.^31^

Analyzing circulating biomarkers is a minimally invasive and promising method to stratify and select patients for different treatments. While the sample size in our study was modest, the observed consistency in biomarker trends suggests potential utility of this multi-analyte panel. The small sample size increases the risk of overfitting in the multivariable Cox model, and the results are therefore interpreted cautiously and should be validated in independent larger cohorts. A strength with the cohort was that all except one patient were treatment-naive, without any influence of previous lines of treatment. Further research is warranted to validate these findings in larger cohorts to explore the interplay between inflammatory signaling and cell proliferation in immune evasion.

### Conclusion

We provide evidence of an additive negative effect of the combination of elevated TKa, CXCL8, HGF, CCL3 and S100A12 on clinical outcome in patients with advanced melanoma treated with anti-PD-1 monotherapy. This multi-biomarker panel comprising proliferation and inflammatory proteins may be utilized as a prognostic tool to more accurately predict the clinical outcome. Taken together, these results suggest that the multi-biomarker panel possesses prognostic value, although further validation in larger cohorts is warranted to confirm its clinical utility.
