# Supplementary material for: Combining circulating inflammatory proteins and thymidine kinase activity to predict survival in melanoma patients receiving immune checkpoint inhibitors
Source: Immunooncol Technol. 2026 Mar 19;30:101595. doi: 10.1016/j.iotech.2026.101595 (PMC13123323; doi:10.1016/j.iotech.2026.101595)

Supplementary Table 1. Table showing the 33 inflammatory proteins significantly differentially expressed between patients with low and high TKa levels at baseline (adjusted p-value < 0.05). Multiple testing correction was performed using the Benjamini-Hochberg method.

Supplementary Table 2. The Table shows the 5-year PFS and OS rates for each individual biomarker, stratified into low and high levels as well as the multi-biomarker panel, stratified according to the number of elevated unfavorable biomarkers at baseline. Cut‑offs were determined based both on the Youden index and median splits. Although only the survival rates based on Youden‑derived cut‑offs for the individual biomarkers are presented in the Table, the multi-biomarker panels were generated using thresholds defined by both the Youden index and median‑based dichotomization.

| **Biomarkers** | **5-year PFS rate (%)** | | **5-year OS rate (%)** | |
| --- | --- | --- | --- | --- |
|  | Youden index | | Youden index | |
| CCL3 |  | |  | |
| low | 33.16 | | 63.16 | |
| high | 22.86 | | 30.00 | |
|  |  | |  | |
| CXCL8 |  | |  | |
| low | 34.14 | | 67.57 | |
| high | 21.43 | | 23.81 | |
|  |  | |  | |
| HGF |  | |  | |
| low | 34.15 | | 67.65 | |
| high | 22.92 | | 29.17 | |
|  |  | |  | |
| S100A12 |  | |  | |
| low | 35.00 | | 69.44 | |
| high | 20.78 | | 22.73 | |
|  |  | |  | |
| TKa |  | |  | |
| low | 34.19 | | 58.14 | |
| high | 15.56 | | 33.33 | |
|  |  | |  | |
|  | Youden index | Median | Youden index | Median |
| Panel |  |  |  |  |
| 0 elevated unfavorable | 42.78 | 38.10 | 83.33 | 83.33 |
| 1 elevated unfavorable | 30.77 | 46.15 | 61.54 | 61.54 |
| 2-3 elevated unfavorable | 22.22 | 22.22 | 33.33 | 50.00 |
| 4-5 elevated unfavorable | 11.11 | 16.00 | 11.11 | 20.00 |


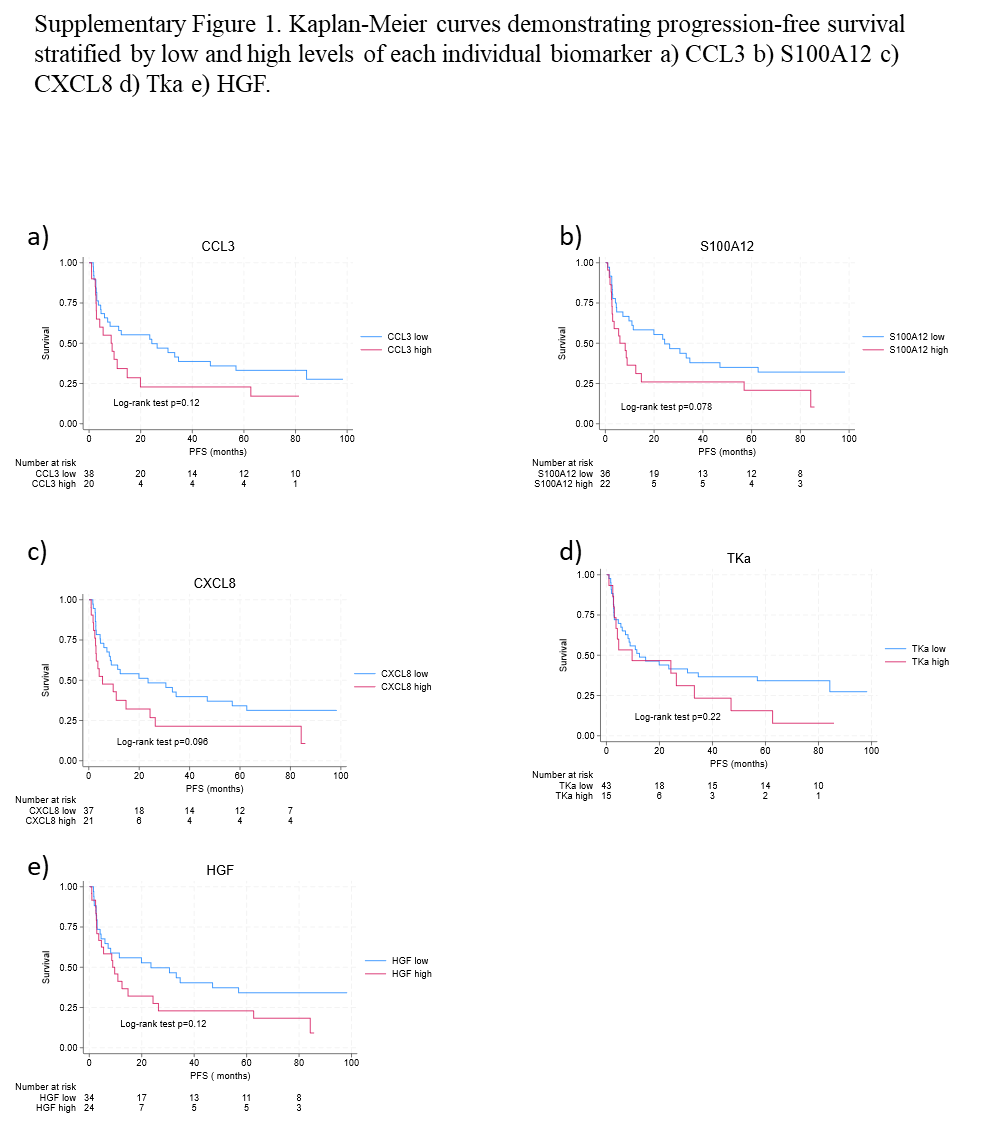


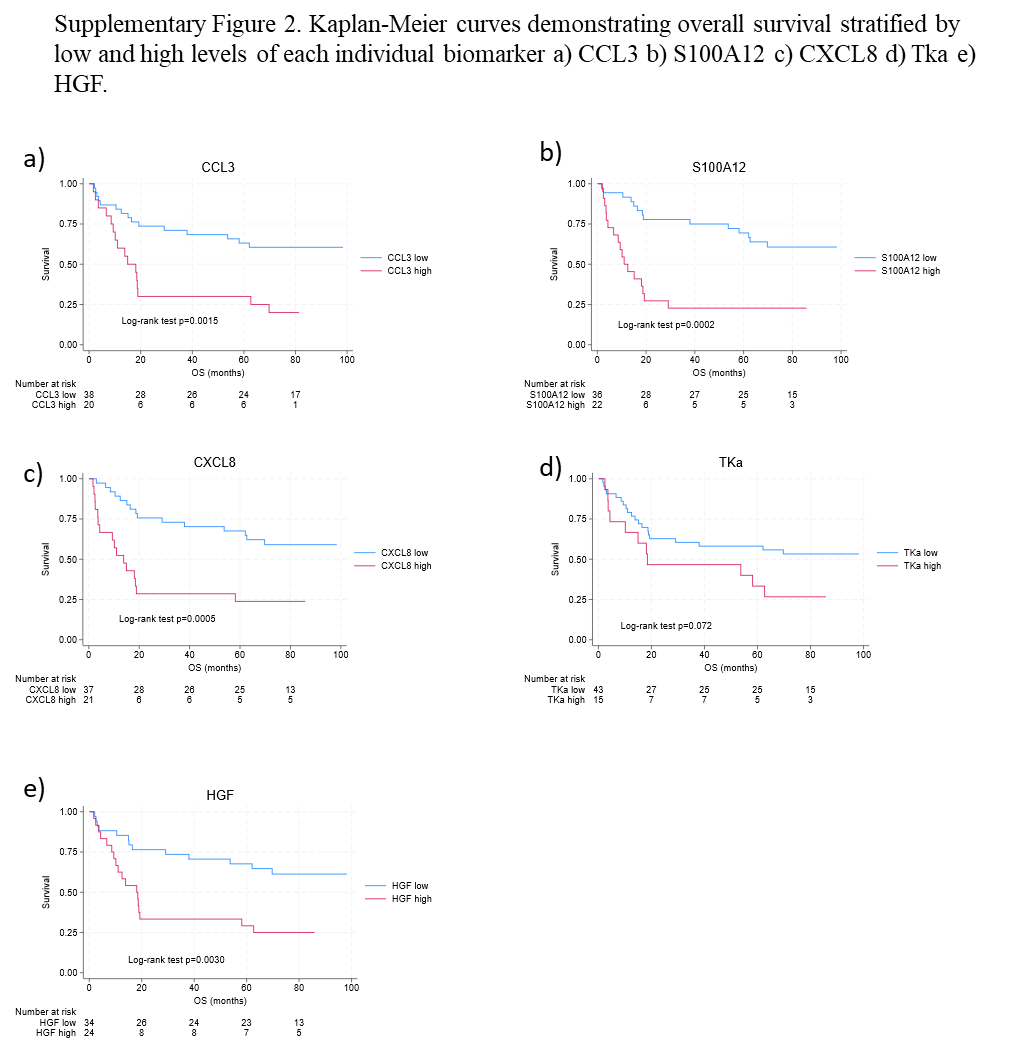


Supplementary Figure 3. Bar plots showing response rates (complete response (CR) and partial response/stable disease (PR/SD) and non-responder (NR)) stratified according to the number of elevated unfavorable biomarkers at baseline.


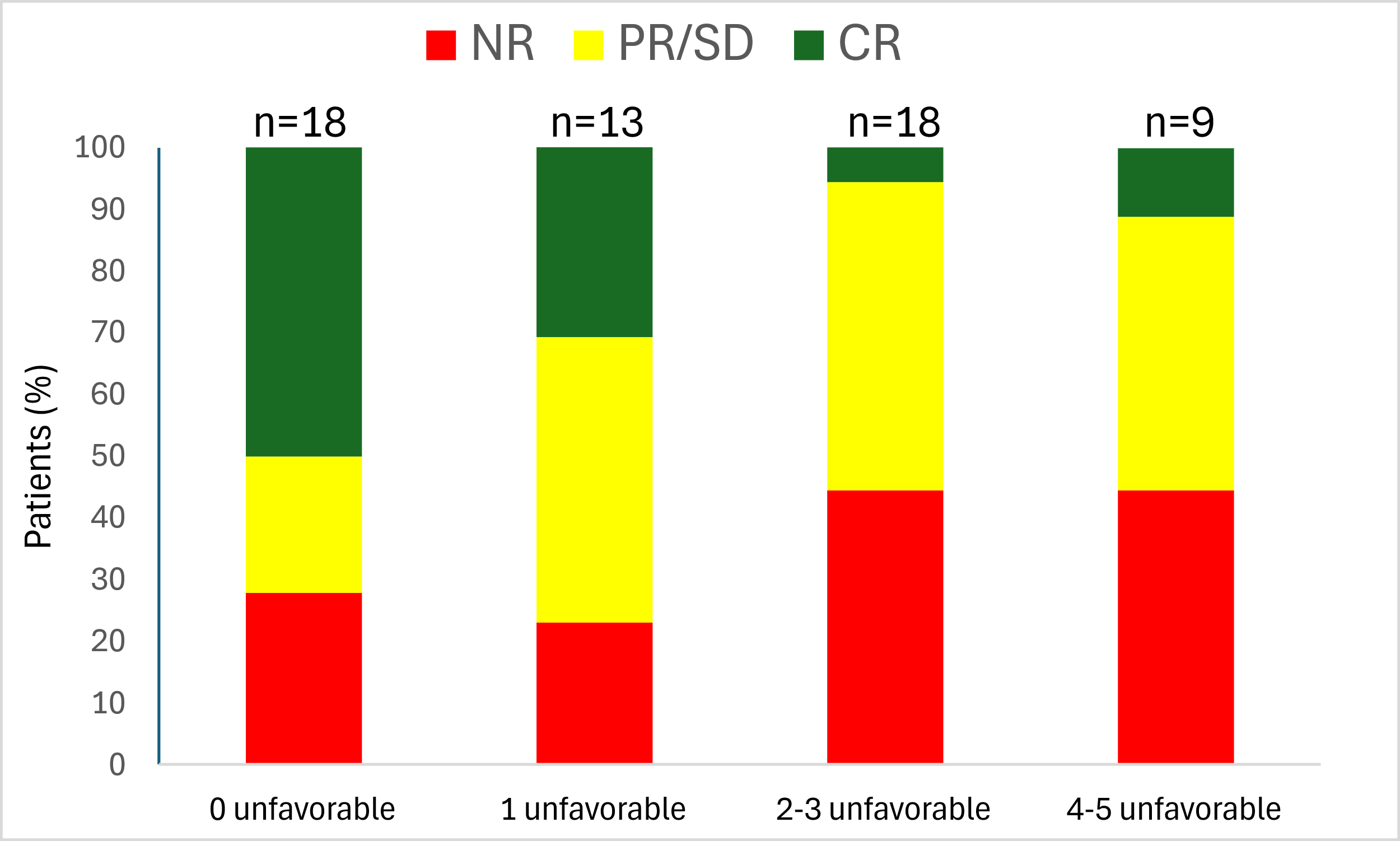

Supplement: Supplementary Material [file mmc1.docx]
